# Gremlin Enhances the Determined Path to Cardiomyogenesis

**DOI:** 10.1371/journal.pone.0002407

**Published:** 2008-06-11

**Authors:** Daisuke Kami, Ichiro Shiojima, Hatsune Makino, Kenji Matsumoto, Yoriko Takahashi, Ryuga Ishii, Atsuhiko T. Naito, Masashi Toyoda, Hirohisa Saito, Masatoshi Watanabe, Issei Komuro, Akihiro Umezawa

**Affiliations:** 1 Department of Reproductive Biology, National Institute for Child Health and Development, Tokyo, Japan; 2 Department of Allergy and Immunology, National Institute for Child Health and Development, Tokyo, Japan; 3 Laboratory for Medical Engineering, Division of Materials Science and Chemical Engineering, Graduate School of Engineering, Yokohama National University, Yokohama, Japan; 4 Department of Cardiovascular Science and Medicine, Chiba University Graduate School of Medicine, Chiba, Japan; Centre de Regulacio Genomica, Spain

## Abstract

**Background:**

The critical event in heart formation is commitment of mesodermal cells to a cardiomyogenic fate, and cardiac fate determination is regulated by a series of cytokines. Bone morphogenetic proteins (BMPs) and fibroblast growth factors have been shown to be involved in this process, however additional factors needs to be identified for the fate determination, especially at the early stage of cardiomyogenic development.

**Methodology/Principal Findings:**

Global gene expression analysis using a series of human cells with a cardiomyogenic potential suggested *Gremlin* (*Grem1*) is a candidate gene responsible for *in vitro* cardiomyogenic differentiation. Grem1, a known BMP antagonist, enhanced DMSO-induced cardiomyogenesis of P19CL6 embryonal carcinoma cells (CL6 cells) 10–35 fold in an area of beating differentiated cardiomyocytes. The Grem1 action was most effective at the early differentiation stage when CL6 cells were destined to cardiomyogenesis, and was mediated through inhibition of BMP2. Furthermore, BMP2 inhibited Wnt/β-catenin signaling that promoted CL6 cardiomyogenesis.

**Conclusions/Significance:**

Grem1 enhances the determined path to cardiomyogenesis in a stage-specific manner, and inhibition of the BMP signaling pathway is involved in initial determination of Grem1-promoted cardiomyogenesis. Our results shed new light on renewal of the cardiovascular system using Grem1 in human.

## Introduction

The critical event in heart formation is commitment of mesodermal cells to a cardiomyogenic fate and their migration into anterolateral regions of the embryo during late gastrulation. In this process, morphogenic movements and cardiac fate determination are regulated by cytokines such as bone morphogenetic proteins (BMPs) [1]–[3], and fibroblast growth factors (FGFs) [4]–[7]. These secreted proteins from neighboring endoderm, ectoderm, and the mesoderm itself, play important roles in induction of cardiac transcription factors [8] and differentiation of cardiomyocytes in amphibians [9] and avians [4]. Cardiomyogenic signals, such as BMPs and FGFs, indeed activate expression of cardiac specific transcriptional factors (Csx/Nkx2.5, Gata4, Mef2c), and these transcriptional factors activate expression of circulating hormones (atrial natriuretic peptide (ANP), brain natriuretic peptide (BNP)), and cardiac specific proteins (myosin heavy chain (MyHC), myosin light chain (MyLC)). Wnt family proteins, cysteine-rich, and secreted glycoproteins, have also been implicated in embryonic development [10], [11], and cardiomyogenesis [12], [13]. In *Drosophila*, ‘*wingless*’, a homologue of vertebrate Wnt is involved in expression of ‘*tinman*’, a *Drosophila* homologue of Csx/Nkx2.5, through ‘*armadillo*’, a *Drosophila* ortholog of β-catenin, and drives heart development [14]. In vertebrates, however, Wnt1/3a, which activates the canonical Wnt/β-catenin signaling pathway leading to stabilization of β-catenin as a downstream molecule through inactivation of glycogen synthase kinase-3β, inhibits cardiomyocytic differentiation from cardiac mesoderm [15]–[18]. Wnt11 promotes cardiac differentiation via the non-canonical pathway in *Xenopus* [12] and murine embryonic cell lines [19]. The secretion of Wnt inhibitors such as ‘Cerberus’, ‘Dickkopf’ and ‘Crescent’ by the anterior endoderm prevents Wnt3a secreted by the neural tube from inhibiting heart formation [15]–[17].

In this study, we performed GeneChip analysis to identify multiple extracellular determinants, such as cytokines, cell membrane-bound molecules and matrix responsible for cardiomyogenic differentiation, and evaluated the statistical significance of differential gene expression by NIA array analysis (http://lgsun.grc.nia.nih.gov/ANOVA/) [20], a web-based tool for microarray data analysis. We found that Grem1 enhances the determined path to cardiomyogenesis in a stage-specific manner, and that inhibition of the BMP signaling pathway is, at least in part, involved in initial determination of Grem1-promoted cardiomyogenesis.

## Results

### GeneChip and statistical analysis

**Figure 1:**
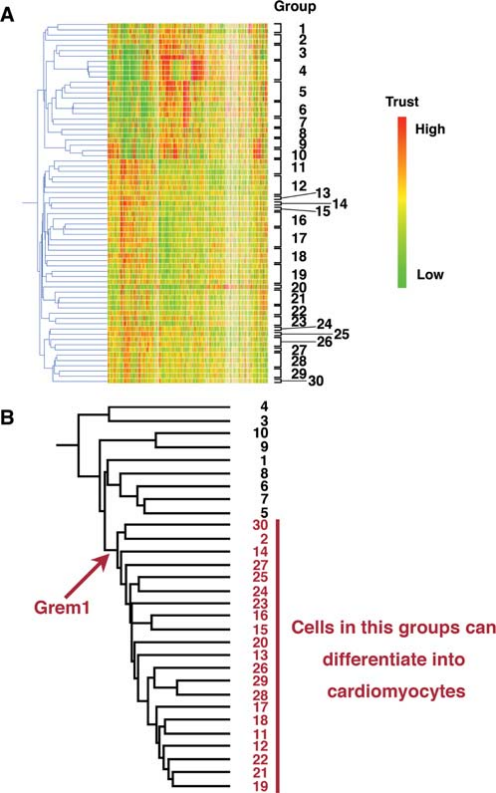
Hierarchical clustering analysis on cultured human cells. (A) Hierarchical clustering analyzed by GeneSpring. Based on gene expression pattern, 69 human cells were clustered into 30 sub-groups. The raw data from the GeneChip analysis are available at the GEO database with accession number GSE8481, GSM41342- GSM41344, and GSM201137- GSM201145. (B) Hierarchical clustering analysis was performed by NIA array (http://lgsun.grc.nia.nih.gov/ANOVA/), using averaged values of 30 sub-groups. Among the 30 groups, 21 groups included cells with a cardiomyogenic potential. To identify genes specific for these groups, hierarchical clustering was employed. *Grem1* was nominated as a cluster-specific cardiomyocyte-promoting gene in cells that could differentiate into cardiomyocytes.

To identify cytokines and transcription factors responsible for cardiomyogenic differentiation, 69 human cells were analyzed, depending on gene expression levels, by GeneSpringGX software, and clustered into 30 groups (Fig. 1A, Table 1). Among the 30 groups, 21 groups included cells with a cardiomyogenic potential (Fig. 1B: red numbers). To identify genes specific for these groups, hierarchical clustering was employed, using the average distance method. Genes with the lowest average expression E(G1) within the cluster that can differentiate into cardiomyocytes and genes with the highest average expression E(G2) outside the cluster were identified, as previously described [20]–[22]. Genes which have E(G1)>E(G2) were estimated, using the False Discovery Rate (FDR<0.05). Grem1 was nominated as a cluster-specific cardiomyocyte-promoting gene in cells that could differentiate into cardiomyocytes following NIA array analysis (Fig. 1B). The gene expression profile reported in this paper has been deposited in the Gene Expression Omnibus (GEO) database (http://www.ncbi.nlm.nih.gov/geo: accession no. GSE8481, GSM41342- GSM41344, and GSM201137- GSM201145).

**Table 1:** 69 human cells clustered into 30 groups

Group		Title	Description	GSM
1	Normal epithelial cell,primary	NHEK-Neo1	Normal epidermal keratinocyte, neonate, primary	GSM210361
		NHBE-1	Normal bronchial epithelial cell, primary	GSM210362
2	Pulmonary epithelial cell line	A549	Pulmonary epithelial cell line	GSM210363
		BEAS-2B control (6hr)	Bronchial epithelial cell line	GSM210364
3	Lymphocyte	RPMI8226control (6hr)	B cell line	GSM210365
		Raji-1	B cell line	GSM210366
		NK92	NK cell line	GSM210367
4	Myelomonocytic leukemia	U937c	U937 control	GSM210368
		U937h	U937+HRF	GSM210369
		U937ha	U937+HRF+antibody	GSM210370
		U937a	U937+antibody	GSM210371
5	Embryonal carcinoma, cancer	NCR-G3	Embryonal carcinoma, NCR-G3, non-adherent	GSM201141
		NCR-G2NAd	Embryonal carcinoma, NCR-G2, non-adherent	GSM210373
		NCR-G4Ad	Embryonal carcinoma, NCR-G4, adherent	GSM201142
		NCR-G3Ad	Embryonal carcinoma, NCR-G3, adherent	GSM210375
6	ES cell	H1_P43	Undifferentiated hES	GSM41342
		H1-P46	Undifferentiated hES	GSM41343
		H1-P41	Undifferentiated hES	GSM41344
7	Embryonal carcinoma, cancer	NCR-G2Ad	Embryonal carcinoma, NCR-G2, adherent	GSM201140
		NCR-G1	Embryonal carcinoma, NCR-G3, non-adherent	GSM201139
8	Ewing, cancer	NCR-EW2	Ewing, cancer	GSM210378
		NCR-EW3	Ewing, ETV4, cancer	GSM210379
9	Ewing, cancer	GST6	Ewing, POU5F1, cancer	GSM201137
		GST6-extra	Ewing, POU5F1, cancer	GSM210381
10	Ewing, cancer	GST6-5az	Ewing, POU5F1, 5azaC, cancer	GSM201138
		GST6-5az-extra	Ewing, POU5F1, 5azaC, cancer	GSM210383
11	Bone marrow cell, primary	H4-1	Bone marrow cell, primary	GSM201143
		UBT5	Bmi-1, hTERT, bone marrow cell	GSM210385
		UBET7	Bmi-1, E6, hTERT, bone marrow cell	GSM210386
12	Ligament-derived cells	#10	Ligament, primary	GSM210387
	Marrow stromal cells	H10-2Vec	Vector, bone marrow cell	GSM210388
		H10-2TERT	hTERT, bone marrow cell	GSM210389
		H10-2Bmi1	Bmi-1, bone marrow cell	GSM210390
13	Placenta, primary	PL90	Placenta, primary	GSM210391
14	De-differentiated chondrocyte	TdHC1	E6, E7, hTERT, de-differentiated chondrocyte	GSM210392
15	Neural differentiated marrow stromal cell	UET13 Neural differentiation	E7, hTERT, neural differentiation, bone marrow cell	GSM210393
16	Neural differentiated marrow stromal cell	UET13 Neural differentiation1	E7, hTERT, neural differentiation, bone marrow cell	GSM210394
		UET13 Neural differentiation4	E7, hTERT, neural differentiation, bone marrow cell	GSM210395
		UET13 Neural differentiation5	E7, hTERT, neural differentiation, bone marrow cell	GSM210396
17	Cord blood-derived cells	UET13	E7, hTERT, bone marrow cell	GSM210397
		UCB408	Cord blood, primary	GSM210398
		UCB408E6E7-31	E6, E7, umbilical cord blood	GSM210399
	Adipocyte cell, primary	HAdPC1(5/21)	HAdpc1E6E7TERT28	GSM210400
18	Marrow mesenchymal cell, primary	UEET12	E6, E7, hTERT, bone marrow cell	GSM210401
		UEE16	E6, E7, bone marrow cell	GSM210402
		EPC hTERT+1	E6, E7, hTERT, endometrial cell	GSM201144
19	Cord blood, primary	UCB302	Cord blood, primary	GSM210382
		UCB302-D7	Cord blood, primary	GSM210405
		UCB302TERT	hTERT, cord blood	GSM210406
		UET9	E7, hTERT, bone marrow cell	GSM210407
20	Cord blood, primary	UCB408E7-32	E7, hTERT, cord blood	GSM210408
21	Fetal fibroblast, primary	HFDPC cont.	Normal follicular dermal papillar cell, primary	GSM210409
		PL112	Placenta, primary	GSM210410
		HF7-3	Fetal fibroblast, primary	GSM210411
22	Bone marrow cell, primary	3F0664	Bone marrow cell (commercial item), primary	GSM201145
		BM-MSC	Bone marrow-derived mesenchymal stem cells	GSM38627
23	ES cell-derived mesenchymal cell	H1 clone 2	ES cell-derived mesenchymal precursor	GSM38628
		H9 clone 1	ES cell-derived mesenchymal precursor	GSM38629
24	Endometrial cell	EPC100	E6, E7, hTERT, endometrial cell	GSM210413
25	Bone marrow cell, primary	Yub10F	Bone marrow cell, primary	GSM210414
26	Endometrial cell	EPC hTERT+2	E6, E7, hTERT, endometrial cell	GSM210415
		EPC Control	E6, E7, hTERT, endometrial cell	GSM210416
27	Endometrial cell	EPC214	E6, E7, hTERT, endometrial cell	GSM210417
28	Menstruation blood-derived mesenchymal cell, primary	#E4	Menstruation blood, primary	GSM210418
		#E4HRF	Menstruation blood, HRF treatment, primary	GSM210419
		#E5HRF	Menstruation blood, HRF treatment, primary	GSM210420
29	Menstruation blood-derived mesenchymal cell, primary	#E6	Menstruation blood, primary	GSM210421
		#E6HRF	Menstruation blood, HRF treatment, primary	GSM210422
30	Menstruation blood-derived mesenchymal cell, primary	#E5	Menstruation blood, primary	GSM210423

### Cardiomyogenic differentiation of CL6 cells with Grem1 and DMSO

**Figure 2:**
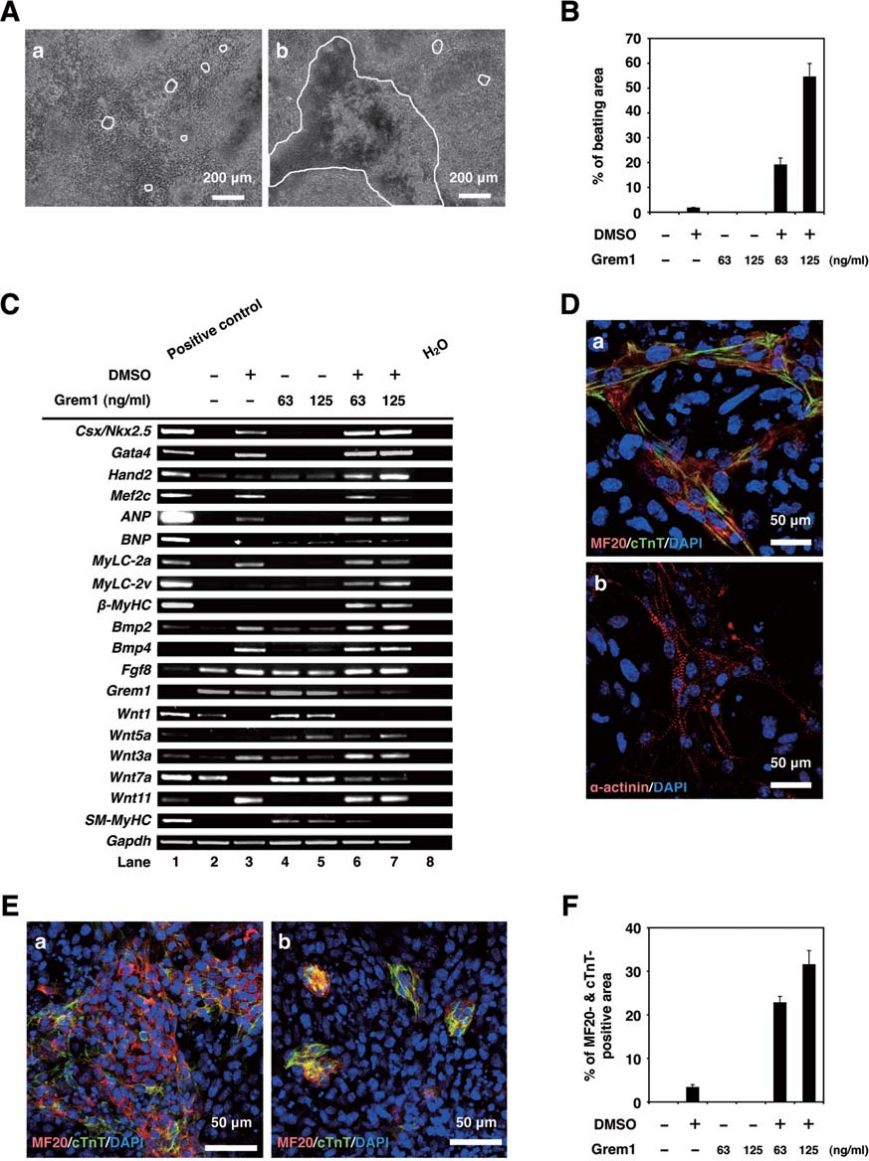
Grem1 enhanced cardiomyogenic differentiation in DMSO-induced CL6 cells. (A) Phase contrast micrograph of CL6 cells with exposure to DMSO alone (a), Grem1 (125 ng/ml) and DMSO (b) for 14 days. The medium, including Grem1 and DMSO, was changed every day. CL6 cells exhibited apparent spontaneous beating between days 9–11. Beating CL6 cell colonies are outlined by white lines. (B) Percentage of beating area in differentiated CL6 cells. CL6 cell treated with Grem1 (125 ng/ml) and DMSO exhibited the strongest contraction. (C) RT-PCR analysis of the genes encoding cardiac-specific transcriptional factors (*Csx/Nkx2.5*, *Gata4*, *Mef2c*, *Hand2*), circulating hormone (*ANP*, *BNP*), cardiac-specific proteins (*MyLC-2a*, *MyLC-2v*, *β-MyHC*), cytokines (*Bmp2*, *Bmp4*, *Fgf8*, *Grem1*, *Wnt1*, *Wnt3a*, *Wnt5a*, *Wnt7a*, *Wnt11*), *SM-MyHC*, and *Gapdh* (From top to bottom). Mouse total heart RNA for the *Csx/Nkx2.5*, *Gata4*, *Mef2c*, *Hand2*, *ANP*, *BNP*, *MyLC-2a*, *MyLC-2v*, *β-MyHC*, *Bmp2*, *Bmp4*, *Grem1*, *Wnt11*, *SM-MyHC*, and *Gapdh* genes, mouse embryonic stem cell RNA for the *Fgf8* gene, and mouse total skeletal muscle RNA for the *Wnt1*, *Wnt3a*, *Wnt5a*, and *Wnt7a* genes were used for positive controls. H_2_O (without RNA) served as a negative control. (D) Immunocytochemistry of CL6 cells 14 days after exposure to Grem1 (125 ng/ml) and DMSO with MF20 and cTnT (a), and α-actinin (b). Cell nuclei are stained with DAPI. Clear striations are evident. (E) Immunocytochemistry of CL6 cells 14 days after exposure to Grem1 and DMSO with cardiac troponin T (cTnT) and sarcomeric myosin (MF20). CL6 cells treated with Grem1 (125 ng/ml) and DMSO (a), and DMSO alone (b) stained positive for cTnT and MF20. Untreated CL6 cells, i.e. not exposed to Grem1(125 ng/ml) or DMSO, stained negative for cTnT and MF20. Cell nuclei were stained with DAPI. (F) Percentage of MF20- and cTnT-double positive area.

To investigate cardiomyogenic activity of Grem1, P19CL6 embryonal carcinoma cells (CL6 cells) were used for assessment of *in vitro* cardiomyogenic differentiation, since CL6 cells are reproducibly and stably induced into beating cardiomyocytes by DMSO (Fig. 2Aa) [23]. CL6 cells did not differentiate following exposure to Grem1 alone at concentrations of 63 or 125 ng/ml for 14 days (Fig. 2B). However, Grem1 dramatically promotes DMSO-induced cardiomyogenic differentiation at a concentration of 63 and 125 ng/ml; Grem1 (125 ng/ml) especially increased DMSO-induced cardiomyogenic differentiation of CL6 cells as assessed by beating area (Fig. 2Ab and B) (Movie S1 and S2, http://1954.jukuin.keio.ac.jp/umezawa/kami/index.html).

### RT-PCR of differentiated or undifferentiated CL6 cells

To investigate gene expression as well as morphological analysis, i.e. beating, during cardiomyogenic differentiation, RT-PCR analysis was performed to detect expression of cardiomyocyte-specific/associate transcription factors, and structural genes (Fig. 2C). Genes encoding *Csx/Nkx2.5*, *Gata4*, *Hand2*, *Mef2c*, *ANP*, *BNP*, *MyLC-2a*, *MyLC-2v*, and *β-MyHC* were up-regulated during cardiomyogenic differentiation of CL6 cells treated with Grem1 and DMSO (Fig. 2C: lanes 6, 7 versus lane 3). Triplicate independent experiments confirmed the concentration-dependent Grem1 action on cardiomyogenic differentiation. The cardiomyocyte-specific genes (*Csx/Nkx2.5*, *Gata4*, *MyLC-2a*, *MyLC-2v*) expression level of CL6 cells treated with DMSO and Grem1 (63 and 125 ng/ml) were also the same as or higher than that of DMSO-induced CL6 cells by semi-quantitative RT-PCR (Figure S1).

### Immunocytochemistry of differentiated or undifferentiated CL6 cells

To examine CL6 cells for expression of cardiomyocytic protein, immunocytochemical analysis was performed. CL6 treated with Grem1 (125 ng/ml) and DMSO exhibited clear striation with immunostain using anti-cTnT or anti-α-actinin (Fig. 2Da and b). The MF20- and cTnT-positive cells after exposure to Grem1 and DMSO formed clusters (Fig. 2Ea), compared with the cells after exposure to DMSO alone (Fig. 2Eb). CL6 cells treated with Grem1 alone were negative for MF20 and cTnT, but became positive for both markers following exposure to Grem1 (63 and 125 ng/ml) and DMSO (Fig. 2F). The beating area (Fig. 2B) showed a tendency similar to the MF20- and cTnT-positive area (Fig. 2F), thus there were positive correlations between them.

### Grem1 and DMSO were most effective at the early stage (days 1–3) of CL6 differentiation

**Figure 3:**
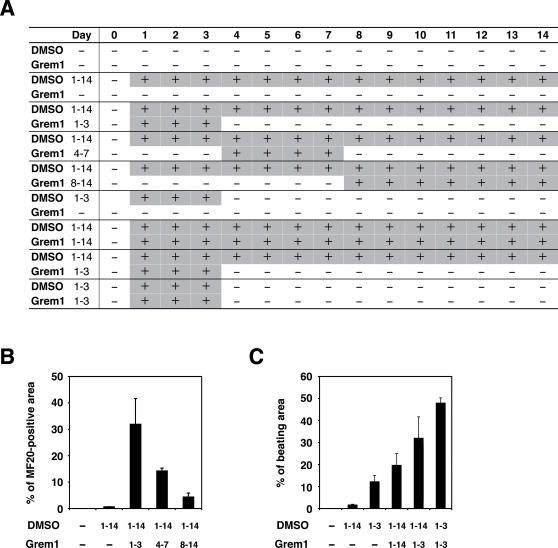
Percentage of myogenic differentiation by period of treatment with Grem1 in CL6 cells. (A) Protocol for treatment of Grem1 and DMSO. CL6 cells were passaged at 1.8×10^5^ cells in 6-well plate on Day 0. CL6 cells were exposed to Grem1 (125 ng/ml) and/or DMSO on the indicated day. Day when the cells were exposed to the inducers is shown by “+” (in gray cells for clarity). The medium including Grem1 and DMSO was changed every day. On day 14, the cells were immunocytochemically stained with MF20 antibody. (B) Myogenic differentiation of CL6 cells was estimated by sarcomeric myosin (MF20)-positive area. CL6 cells were treated with Grem1 (125 ng/ml) and DMSO for the indicated days. (C) Myogenic differentiation of CL6 cells was estimated by beating area. CL6 cells treated with DMSO and Grem1 (125 ng/ml) were incubated at indicated days.

**Figure 4:**
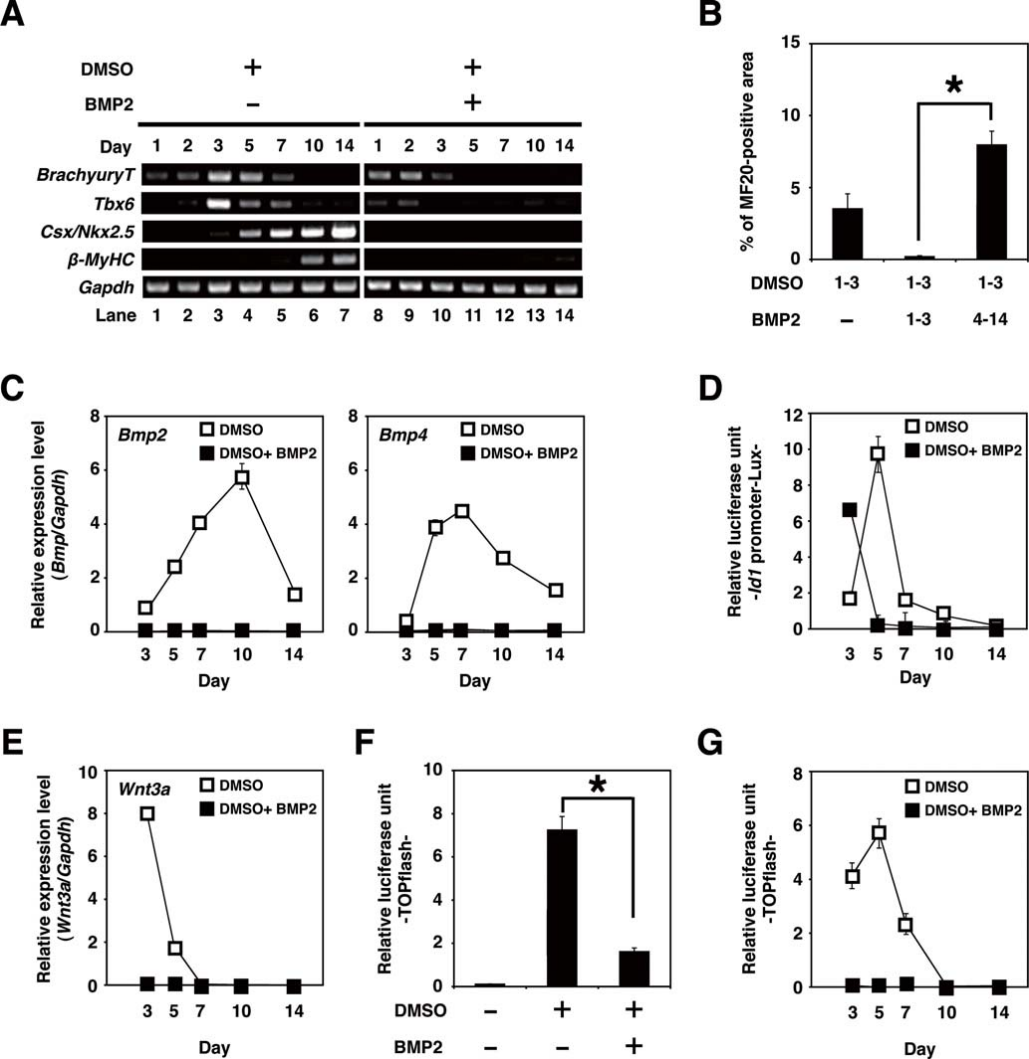
Cardiomyogenic differentiation in CL6 cells (days 1–3) is inhibited by BMP2. (A) RT-PCR analysis of the gene encoding *BrachyuryT*, *Tbx6*, cardiac-specific transcriptional factor (*Csx/Nkx2.*5), cardiac-specific protein (*β-MyHC*), and *Gapdh* (From top to bottom) of CL6 cells treated with DMSO alone, or DMSO and BMP2 (100 ng/ml) for the first 3 days (days 1–3). The medium, including BMP2 and DMSO, was changed every day. (B) Percentage of MF20-positive area. Immunocytochemistry was carried out on CL6 cells 14 days after cells had been exposed to DMSO and BMP2 (100 ng/ml) for the first 3 days (days 1–3). The asterisk indicates a significant statistical difference (*P*<0.05). (C) Quantitative real-time RT-PCR analysis of the gene encoding *Bmp2* (left), and *Bmp4* (right) in CL6 cells treated with DMSO alone (open square), or DMSO and BMP2 (100 ng/ml) (closed square) for the first 3 days (days 1–3). (D) BMP signaling activity of CL6 cells treated with DMSO alone (open square), or DMSO and BMP2 (100 ng/ml) (closed square) for the first 3 days (days 1–3) were determined by luciferase activity analysis using *Id1* promoter-Lux (a firefly luciferase reporter plasmid driven by the Id1 binding sites), pRL-CMV as co-transfected control, and Dual luciferase reporter assay system. Relative luciferase unit of the CL6 cells untreated with inducers at day 3 is regarded as 0.1 (data not shown). (E) Quantitative real-time RT-PCR analysis of the gene encoding *Wnt3a* in CL6 cells treated with DMSO alone (open square), or DMSO and BMP2 (100 ng/ml) (closed square) for the first 3 days (days 1–3). (F) Wnt/β-catenin signaling activity of CL6 cells 48 h after exposure to DMSO, or DMSO and BMP2 (100 ng/ml) was determined by luciferase activity analysis using TOPflash (a firefly luciferase reporter plasmid driven by two sets of three copies of the TCF binding site and herpes simple virus thymidine kinase minimal promoter), pRL-CMV as co-transfected control, and Dual luciferase reporter assay system. Relative luciferase unit of the CL6 cells untreated with inducers is regarded as 0.1. The asterisk indicates a significant statistical difference (*P*<0.05). (G) Timeframe of Wnt/β-catenin signaling activity in CL6 cells treated with DMSO alone (open square), or DMSO and BMP2 (100 ng/ml) (closed square) for the first 3 days (days 1–3). Relative luciferase unit of the CL6 cells untreated with inducers at day 3 is regarded as 0.1 (data not shown).

To determine if Grem1 (125 ng/ml) functions during the early or the late stage of differentiation, CL6 cells were treated with Grem1 for different time periods (Fig. 3A). Grem1 and DMSO were most effective on CL6 differentiation at 1–3 days (Fig. 3B, C) as assessed by percentages of MF20-positive area and beating area. Since Grem1 inhibits BMPs through direct binding [24], we hypothesized that BMP signaling is inhibitory to CL6 cardiomyogenesis during days 1–3. To confirm this hypothesis, RT-PCR analysis was performed to determine expression of the early mesodermal marker (*BrachyuryT* and *Tbx6*), cardiomyocyte-specific transcription factors (*Csx/Nkx2.5*), structural genes (*β-MyHC*), and *Gapdh* (Fig. 4A). DMSO induced the *BrachyuryT* and *Tbx6* genes, and their expressions peaked at 3 days and then decreased; BMP2 down-regulated expression of these genes at 3–7 days. The *Csx/Nkx2.5* and *β-MyHC* genes started to be expressed at days 3 and 5, respectively, and their expression increased up to 14 days, at which time the timeframe analysis was terminated. BMP2 clearly inhibited expression of the *Csx/Nkx2.5* and *β-MyHC* genes (Fig. 4A, lanes 1–7 versus lanes 8–14).

To examine cardiomyogenic differentiation, immunocytochemical analysis was performed on CL6 cells treated with the inducers. CL6 cells treated with DMSO and BMP2 for the first 3 days were negative for sarcomeric myosin (MF20) at 14 days, but became positive for sarcomeric myosin, following exposure to DMSO alone during days 1–3 (Fig. 4B). To determine if DMSO induces BMP production in CL6 cells, expression levels of *Bmp2* and *Bmp4* were determined by quantitative real-time RT-PCR analysis (Fig. 4C). DMSO clearly induced the *Bmp2* and *Bmp4* genes, and DMSO-induction was inhibited by BMP2 protein. The expression level of *Bmp2* was highest during days 7–10 (Fig. 4C: *Bmp2*) in DMSO-induced CL6 cells, and that of *Bmp4* was highest during days 5–7 (Fig. 4C: *Bmp4*).

To investigate BMP signaling on cardiomyogenic differentiation, we used the *Id1* promoter-Lux plasmid that includes the luciferase gene driven by the *Id1* promoter, known as a BMP target promoter (Fig. 4D). DMSO increased BMP signaling activity that peaked at 5 days (Fig. 4D, open square). BMP2 protein increased BMP signaling activity at 3 days (Fig. 4D, closed square), but lost BMP signaling activity at 5 days and later, implying that this loss of BMP signaling leads to lack of cardiomyogenic induction.

Since Wnt/β-catenin signaling is involved in CL6 cardiomyogenesis [23], [25], we hypothesized that the BMP effect on CL6 cardiomyogenesis is mediated through Wnt/β-catenin signaling. Expression of Wnt3a, an activator of canonical Wnt signaling, was indeed detected in CL6 cells exposed to DMSO, and BMP2 significantly down-regulated *Wnt3a* expression at day 3 (Fig. 4E). By using the TOPflash plasmid [23] which includes the luciferase gene driven by two sets of three copies of the TCF recognition site, Wnt/β-catenin signaling was assessed to investigate the effect of BMP2. Wnt/β-catenin signaling activity increased at 48 h after treatment with DMSO. Activity was increased by DMSO treatment but decreased by BMP2 (Fig. 4F). Time course analysis revealed that Wnt/β-catenin activity peaked at 5 days after DMSO treatment, and decreased thereafter (Fig. 4G). BMP2 inhibited DMSO-induced Wnt/β-catenin activity throughout the experimental period (up to 14 days). These results imply that BMP signaling inhibits CL6 cardiomyogenesis at the early stage through inhibition of Wnt/β-catenin signaling.

## Discussion

**Figure 5:**
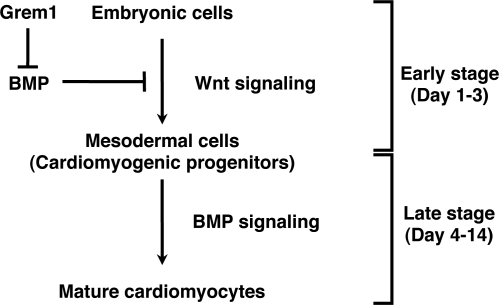
Grem1-accelerated CL6 cardiomyogenesis through regulation of BMP- and Wnt/β-catenin-signaling pathways. CL6 embryonic cells start to differentiate into mesodermal cells through Wnt/β-catenin signaling pathway at the early stage (days 1–3), and mesodermal CL6 cells differentiate into mature cardiomyocytes by BMP pathway at the late stage (days 4–14). Grem1 accelerates DMSO-induced cardiomyogenesis through inhibition of the BMP-signaling pathway.

Our bioinformatics study using the results from the global gene expression analysis of human cells (GSM412342-41344 and GSM201137-201145 at http://www.ncbi.nlm.nih.gov/geo) nominated Grem1 as a candidate gene that may participate in cardiomyogenesis. By using CL6 embryonic cells as a model of cardiomyogenesis, we obtained two major findings: the first is that Grem1 enhanced cardiomyogenic differentiation of DMSO-induced CL6 cells at the early stage; the second is that Wnt/β-catenin and BMP signaling activity had developmental stage-specific effects on cardiomyogenesis (Fig. 5). Wnt/β-catenin activity at the early stage enhanced embryonic cell differentiation into cardiomyocytes, while suppressing this activity by BMP2 or BMP4 proteins as reported in the avian embryo [26]. In contrast, BMP signaling activity in the late stage enhanced cardiomyocytic differentiation. Grem1 regulated the stage-specific Wnt/β-catenin and BMP signaling activity on cardiomyogenesis.

Many studies have indicated that Grem1 is involved in cell differentiation and development, such as osteogenesis [27], lung morphogenesis [28], myogenesis [29], and limb formation [30], through inhibition of BMP2 and BMP4. Grem1-null mice show intact heart development, despite impairment of lung and kidney [31], and therefore Grem1 is considered not to be involved in cardiogenesis, or supplementary factors such as Noggin [32], with a similar function, may compensate Grem1 during development. Grem1 had an enhancing or promoting activity in *in vitro* cardiomyogenesis, as is the case with platelet-derived growth factor as a promoter of cell growth [33]. In this study, Grem1 was involved in cardiomyocyte differentiation. However Grem1 alone could not induce cardiomyocytic differentiation of CL6 cells in the absence of DMSO (Fig. 2C and F), suggesting that Grem1 is solely a promoter of cardiomyogenic differentiation. One of the possible mechanisms for Grem1-enhanced cardiomyogenesis at the early stage is inhibition of the BMP signaling pathway [3]. Alternatively, Grem1-enhanced cardiomyogenesis may be mediated through proliferation of cardiac progenitor cells, as is the case of myogenic progenitor proliferation by Grem1 [34], and this possibility is supported by an increased number of sarcomeric myosin-positive CL6 cardiomyocytes (Fig. 2E and F).

The stage specificity of the Grem1 effect is possibly correlated with the biphasic and antagonistic effect of Wnt/β-catenin signaling on cardiomyogenesis, depending on the stage of development *in vitro* [25] and *in vivo* [35]. CL6 cells differentiated into cardiomyocytes via mesodermal induction by the Wnt/β-catenin signaling pathway at the early stage, and CL6 mesodermal cells differentiated into cardiomyocytes induced by BMP2 at the late stage. It is conceivable that embryonic cells, such as CL6 cells and ES cells, differentiate into cardiomyocytes by inhibiting BMP signaling via putative “mesodermal cells” or “cardiomyogenic progenitors”, or differentiation stages corresponding to these cells (Fig. 5, Figure S2). The early stage process from embryonic cells to mesodermal cells was mediated via Wnt/β-catenin signaling (Fig. 4F, G), and was assessed by expression of *BrachyuryT* and *Tbx6* genes (Fig. 4A), which are target genes for Wnt/β-catenin signaling [36]. BMP signaling antagonizes the cell fate-inducing activity of Wnt/β-catenin [37]. When embryonic cells or cardiomyogenic progenitors are induced to become mature cardiomyocytes by cytokines and growth factors, we must be careful with respect to the stage of cell differentiation because of the biphasic differential action of the factors which are dependent upon the differentiation stage.

In conclusion, we have demonstrated that Grem1 enhances the commitment or determined path to cardiogenic differentiation of CL6 teratocarcinoma cells. Apart from a role in development, Grem1 may serve a clinical use in cardiology, like granulocyte colony-stimulating factor that accelerates production of granulocytes in both peripheral blood and bone marrow. Nomination of *Grem1* as a cardiomyogenic factor is based on hierarchical clustering analysis using global gene expression data of human cells. This bioinformatics approach may be useful for identifying morphogens/factors that can induce differentiation of other cell types/tissues/organs.

## Materials and Methods

### GeneChip analysis

GeneChip analysis was performed (Fig. 1A, Table 1) as previously described [38]. Human genome-wide gene expression was examined with the Human Genome U133A Probe array (GeneChip; Affymetrix), which contains the oligonucleotide probe set for approximately 23,000 full-length genes and expressed sequence tags, according to the manufacturer's protocol (Expression Analysis technical manual and GeneChip Small Sample Target Labeling Assay version 2 technical note [http://www.affymetrix.com/support/technical/index.affx]). Data analysis was performed by the GeneChip Operation System (Affymetrix) and GeneSpringGX software (Silicon Genetics). To normalize the staining intensity variations between chips, the average difference values for all genes on a given chip were divided by the median of all measurements on that chip. Hierarchical-clustering analysis was performed using a minimum distance value of 0.001, a separation ratio of 0.5, and the standard definition of the correlation distance.

### Cell culture and differentiation

CL6 cells were grown on 100 mm dishes (Becton Dickinson) in α-MEM (Gibco) supplemented with 10% fetal bovine serum (FBS) (JRH Bioscience, Inc.), penicillin, and streptomycin, and were maintained in a 5% CO_2_ atmosphere at 37°C. To induce differentiation, CL6 cells were plated at a density of 1.8×10^5^ cells in a 6-well plate (Becton Dickinson) or gelatin-coated 35 mm glass base dishes (IWAKI) with α-MEM containing Grem1 (63 or 125 ng/ml: R&D system) and/or 1% dimethyl sulfoxide (DMSO) for 14 days. Recombinant human bone morphogenetic protein-2 (BMP2) was purchased from R&D systems.

### Reverse transcriptase-PCR (RT-PCR) and quantitative real-time RT-PCR analysis

Total RNAs were extracted from differentiated and undifferentiated CL6 cells and mouse embryonic stem (ES) cells with RNeasy minikit and DNase I treatment (QIAGEN). Mouse ES cell (129 strains) RNA, mouse heart total RNA (Clontech) and mouse skeletal muscle/total RNA (UNITECH. Co., Ltd.) were used as a positive control for each primer. Total RNA (2.0 µg each) for RT-PCR was converted to cDNA with Superscript™ III RNase H– reverse transcriptase (Invitrogen), according to the manufacturer's manual. PCR conditions were optimized and linear amplification range was determined for each primer by varying annealing temperature and cycle number. PCR products were identified by positive control size. RT-PCR was performed using the primers of the genes of cardiac specific transcription factors: *Csx/Nkx2.5*, *Gata4*, *Mef2c*, *Hand2*; circulating hormone: *ANP*, *BNP*; cardiac structural proteins: *β-MyHC*, *MyLC-2a*, *MyLC-2v*; cytokines: *Bmp2*, *Bmp4*, *Fgf8*, *Grem1*, *Wnt1*, *Wnt3a*, *Wnt5a*, *Wnt7a*, *Wnt11*; smooth muscle structural protein: smooth muscle-myosin heavy chain (*SM-MyHC*); the early mesodermal marker: *BrachyuryT, T-box6* (*Tbx6*); and *Gapdh* as control. PCR was performed with exTaq DNA polymerase and exTaq PCR buffer (TaKaRa) or LATaq DNA polymerase and GC buffer I (TaKaRa) for 25 or 30 cycles, with each cycle consisting of 95°C for 30 s, 50°C, 55°C, 60°C or 65°C for 45 s, and 72°C for 45 s, with an additional 5 min incubation at 72°C after completion of the final cycle. PCR primers for the genes of *Csx/Nkx2.5*, *Gata4*, *Mef2c*, *Hand2*, *ANP*, *BNP*, *β-MyHC*, *MyLC-2a*, *MyLC-2v*, *Bmp2*, *Bmp4*, *Fgf8*, *Grem1*, *Wnt1*, *Wnt3a*, *Wnt5a*, *Wnt7a*, *Wnt11*, *SM-MyHC*, *BrachyuryT*, *Tbx6*, and *Gapdh* (Table S1a) were obtained from Mouse Genome Informatics (http://www.informatics.jax.org/). The PCR products were size-fractionated by 2% agarose gel electrophoresis.

Quantitative real-time RT-PCR was performed on an ABI Prism 7700 Sequence Detection System (Applied Biosystems), using 100 ng of cDNA in 25 µl reaction volume with 10 nmol/l of each primer, and 12.5 µl SYBR Green Realtime PCR Master Mix (TOYOBO). PCR primers for the genes of *Bmp2*, *Bmp4*, *Wnt3a*, and *Gapdh* (Table S1b) were obtained from PrimerBank (http://pga.mgh.harvard.edu/primerbank/index.html). Calculations were automatically performed by ABI software (Applied BioSystems).

### Immunocytochemistry

A laser confocal microscope (LSM510, Zeiss) was used for immunocytochemical analysis. Differentiated and undifferentiated CL6 cells were fixed with 4% paraformaldehyde (Wako) for 5 min at 4°C and treated with 0.1% triton X-100 (Sigma) in PBS for 20 min at room temperature, then incubated for 20 min at room temperature in a protein-blocking solution consisting of PBS supplemented with 5% normal goat serum (DakoCytomation). These CL6 cells were then incubated overnight with primary antibody monoclonal anti-sarcomeric myosin antibody (MF20, mouse IgG_2b_ isotype, 1 mg/ml, University of Iowa Hybridoma Bank) and Troponin T, and Cardiac Isoform Ab-1 clone 13-11 (cTnT, mouse IgG_1_ isotype, 1∶300, Lab Vision Corp), or the monoclonal anti-α-actinin (SARCOMERIC) CLONE EA-53 (α-actinin, mouse IgG_1_ isotype, 1∶300, Sigma) in PBS at 4°C. The cells were extensively washed in PBS and incubated at room temperature with Alexa Fluor 568-conjugated goat anti-Mouse IgG_2b_ (anti-MF20) (Molecular Probe; diluted 1∶300), Alexa Fluor 488-conjugated goat anti-mouse IgG_1_ (anti-cTnT) (Molecular Probe; diluted 1∶300), Alexa Fluor 546-conjugated goat anti-mouse IgG(H+L) (anti-α-actinin) (Molecular Probe; diluted 1∶300), and nuclei were counterstained with 4′, 6-diamidino-2-phenylindole (DAPI) (Wako; diluted 1∶300) for 45 min. To prevent fading, cells were then mounted in DakoCytomation Fluorescent Mounting Medium (DakoCytomation).

### Transfection and luciferase assays

Cells (8.0×10^5^) seeded and cultured in 60 mm dishes (Becton Dickinson) were transfected 18 h after plating using Lipofectamine 2000 (Invitrogen) and PLUS reagent (Invitrogen) in Opti-MEM (Gibco). Transfection contained 1.0 µg of TOPflash plasmid (Upstate Biotechnology) for measurement of Wnt/β-catenin activity, or 5.0 µg of the *Id1* promoter-Lux plasmid (provided by Dr Imamura and Dr Miyazono) for measurement of BMP-induced *Id1* gene transcription, and 0.5 µg of pRL-CMV (Promega) as co-transfected control. Medium containing 10% FBS was changed 3 h after transfection and transfected cells (1.8×10^5^) were re-seeded in 6-well plates 24 h after transfection. After 18 h, CL6 cells were induced with BMP2 (100 ng/ml) and DMSO. CL6 cells were prepared for luciferase activity analysis using Dual luciferase reporter assay system (Promega).

### Area calculation

The regions of interest (beating area, immunostaining area) were defined in Photoshop (Adobe systems) using the ‘magic wand’ tool. The total numbers of pixels identified were then counted using the histogram function. At least five different fields were measured for each dish.

### Statistical analysis

Results, shown as the mean±SE, were compared by ANOVA followed by Scheffé's test, with *P*<0.05 considered significant.

## Supporting Information

Figure S1 A semi-quantitative RT-PCR of cardiomyocyte-specific genes. To investigate expression level of cardiomyocyte-specific genes (Csx/Nkx2.5, Gata4, MyLC-2a, and MyLC-2v), a semi-quantitative RT-PCR was performed from CL6 cells treated with 1% DMSO and the indicated concentration of Grem1 for 14 days. Each RT-PCR product was electrophoresed in 2% agarose gel, and was measured using ImageJ software (http://rsb.info.nih.gov/ij/) to calculate the ratio of each gene to Gapdh. The expression level for each gene is determined relative to that of Gapdh, and expression level in CL6 cells treated with DMSO alone was regarded as 1.0. The relative expression levels were averaged from at least three independent experiments.
(1.04 MB DOC)

Figure S2 Grem1 enhanced cardiomyogenic differentiation of mouse ES cells. Mouse ES cells (NCH1.5, C57BL/6J×129ter/Sv) were cultured on a mouse embryonic fibroblast feeder layer inactivated with 30 Gy γ-irradiation in gelatin-coated 60 mm dishes (Becton, Dickinson). Cells were grown in KnockOut DMEM (Gibco) supplemented with 15% fetal bovine serum (Cell Culture Technologies), 2 mM GlutaMAX (Gibco), 0.1 mM non-essential amino acid (Gibco), 0.1 mM 2-mercaptoethanol (Gibco), penicillin, streptomycin, and 2,000 U/ml mouse leukemia inhibitory factor (LIF) (Chemicon). For cardiomyogenic differentiation, ES cells were exposed to 125 ng/ml Grem1 (R&D systems) for the three days. The cells were then trypsinized and cultured to form embryonic bodies (EBs) from a single cell using a three-dimensional culture system (without LIF) on low cell binding dishes (96-well plate round bottom). This represented day 0 of EB formation. On the next day, the medium was replaced with the same medium without LIF. EBs were re-seeded on gelatin-coated 48-well plates with one EB per well, on day 8 after the start of EB formation. The cardiomyogenic induction was estimated by the beating EB number per total EB number, measured on day 12 under a phase-contrast microscope. Grem1 increased the percentage of beating EBs to 69.2%, as compared with 26.7% in EBs without Grem1 treatment. The numbers in parentheses indicate the EB numbers counted.
(1.27 MB DOC)

Table S1 Primer sequences.
(0.06 MB DOC)

Movie S1 CL6 cells treated with DMSO alone. P19CL6 cells are reproducibly and stably induced into beating cardiomyocytes with DMSO.
(1.66 MB MOV)

Movie S2 CL6 cells treated with Grem1 (125 ng/ml) and DMSO. Grem1 dramatically promotes DMSO-induced cardiomyogenic differentiation of P19CL6 cells at a concentration of 125 ng/ml.
(2.40 MB MOV)
