# Supplementary material for: Gremlin Enhances the Determined Path to Cardiomyogenesis
Source: PLoS One. 2008 Jun 11;3(6):e2407. doi: 10.1371/journal.pone.0002407 (PMC2398777; doi:10.1371/journal.pone.0002407)
Supplement: Figure S1 — A semi-quantitative RT-PCR of cardiomyocyte-specific genes. To investigate expression level of cardiomyocyte-specific genes (Csx/Nkx2.5, Gata4, MyLC-2a, and MyLC-2v), a semi-quantitative RT-PCR was performed from CL6 cells treated with 1% DMSO and the indicated concentration of Grem1 for 14 days. Each RT-PCR product was electrophoresed in 2% agarose gel, and was measured using ImageJ software (http://rsb.info.nih.gov/ij/) to calculate the ratio of each gene to Gapdh. The expression level for each gene is determined relative to that of Gapdh, and expression level in CL6 cells treated with DMSO alone was regarded as 1.0. The relative expression levels were averaged from at least three independent experiments. (1.04 MB DOC) [file pone.0002407.s001.doc]

### **Supporting Information**

**Figure 1S. A semi-quantitative RT-PCR of cardiomyocyte-specific genes**


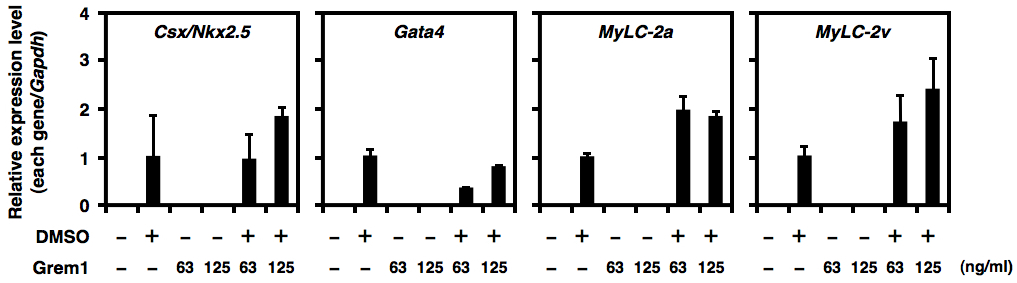


To investigate expression level of cardiomyocyte-specific genes (*Csx/Nkx2.5*, *Gata4*, *MyLC-2a*, and *MyLC-2v*), a semi-quantitative RT-PCR was performed from CL6 cells treated with 1% DMSO and the indicated concentration of Grem1 for 14 days. Each RT-PCR product was electrophoresed in 2% agarose gel, and was measured using ImageJ software (http://rsb.info.nih.gov/ij/) to calculate the ratio of each gene to *Gapdh*. The expression level for each gene is determined relative to that of *Gapdh*, and expression level in CL6 cells treated with DMSO alone was regarded as 1.0. The relative expression levels were averaged from at least three independent experiments.
