# Supplementary material for: Gremlin Enhances the Determined Path to Cardiomyogenesis
Source: PLoS One. 2008 Jun 11;3(6):e2407. doi: 10.1371/journal.pone.0002407 (PMC2398777; doi:10.1371/journal.pone.0002407)
Supplement: Figure S2 — Grem1 enhanced cardiomyogenic differentiation of mouse ES cells. Mouse ES cells (NCH1.5, C57BL/6J×129ter/Sv) were cultured on a mouse embryonic fibroblast feeder layer inactivated with 30 Gy γ-irradiation in gelatin-coated 60 mm dishes (Becton, Dickinson). Cells were grown in KnockOut DMEM (Gibco) supplemented with 15% fetal bovine serum (Cell Culture Technologies), 2 mM GlutaMAX (Gibco), 0.1 mM non-essential amino acid (Gibco), 0.1 mM 2-mercaptoethanol (Gibco), penicillin, streptomycin, and 2,000 U/ml mouse leukemia inhibitory factor (LIF) (Chemicon). For cardiomyogenic differentiation, ES cells were exposed to 125 ng/ml Grem1 (R&D systems) for the three days. The cells were then trypsinized and cultured to form embryonic bodies (EBs) from a single cell using a three-dimensional culture system (without LIF) on low cell binding dishes (96-well plate round bottom). This represented day 0 of EB formation. On the next day, the medium was replaced with the same medium without LIF. EBs were re-seeded on gelatin-coated 48-well plates with one EB per well, on day 8 after the start of EB formation. The cardiomyogenic induction was estimated by the beating EB number per total EB number, measured on day 12 under a phase-contrast microscope. Grem1 increased the percentage of beating EBs to 69.2%, as compared with 26.7% in EBs without Grem1 treatment. The numbers in parentheses indicate the EB numbers counted. (1.27 MB DOC) [file pone.0002407.s002.doc]

### **Supporting Information**

**Figure 2S. Grem1 enhanced cardiomyogenic differentiation of mouse ES cells**


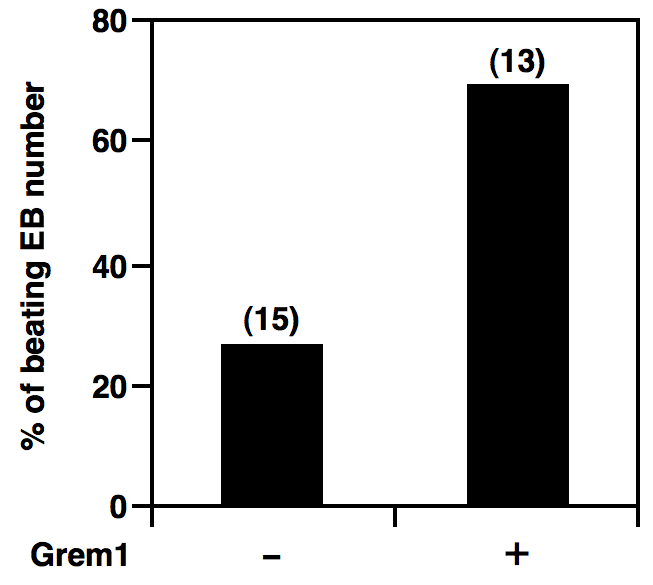


Mouse ES cells (NCH1.5, C57BL/6J x 129ter/Sv) were cultured on a mouse embryonic fibroblast feeder layer inactivated with 30 Gy γ-irradiation in gelatin-coated 60 mm dishes (Becton, Dickinson). Cells were grown in KnockOut DMEM (Gibco) supplemented with 15% fetal bovine serum (Cell Culture Technologies), 2 mM GlutaMAX (Gibco), 0.1 mM non-essential amino acid (Gibco), 0.1 mM 2-mercaptoethanol (Gibco), penicillin, streptomycin, and 2,000 U/ml mouse leukemia inhibitory factor (LIF) (Chemicon). For cardiomyogenic differentiation, ES cells were exposed to 125 ng/ml Grem1 (R&D systems) for the three days. The cells were then trypsinized and cultured to form embryonic bodies (EBs) from a single cell using a three-dimensional culture system (without LIF) on low cell binding dishes (96-well plate round bottom). This represented day 0 of EB formation. On the next day, the medium was replaced with the same medium without LIF. EBs were re-seeded on gelatin-coated 48-well plates with one EB per well, on day 8 after the start of EB formation. The cardiomyogenic induction was estimated by the beating EB number per total EB number, measured on day 12 under a phase-contrast microscope. Grem1 increased the percentage of beating EBs to 69.2%, as compared with 26.7% in EBs without Grem1 treatment. The numbers in parentheses indicate the EB numbers counted.
