# Supplementary material for: Gremlin Enhances the Determined Path to Cardiomyogenesis
Source: PLoS One. 2008 Jun 11;3(6):e2407. doi: 10.1371/journal.pone.0002407 (PMC2398777; doi:10.1371/journal.pone.0002407)
Supplement: Table S1 — Primer sequences. (0.06 MB DOC) [file pone.0002407.s003.doc]

### **Supporting Information**

**Supplemental table 1. Primer sequences**

**a. Primers for RT-PCR**

| **Gene product** | **Sense** | **Anti-sense** |
| --- | --- | --- |
| ***Csx/Nkx2.5*** | TGGCGTCTGGGGACCTGTCTG | GAGTCTGGTCCTGCCGCTGTC |
| ***Gata4*** | TACATGGCCGACGTGGGAGCA | TGGAGTTACCGCTGGAGGCAC |
| ***Hand2*** | TACCAGCTACATCGCCTACCT | TCACTGCTTGAGCTCCAGGG |
| ***Mef2c*** | AGCAAGAATACGATGCCATC | GAAGGGGTGGTGGTACGGTC |
| ***ANP*** | TTGGCTTCCAGGCCATAATTG | AAGAGGGCAGATCTATCGGA |
| ***BNP*** | ATGGATCTCCTGAAGGTGCT | TCTTGTGCCCAAAGCAGCTT |
| ***MyLC-2a*** | CAGACCTGAAGGAGACCT | GTCAGCGTAAACAGTTGC |
| ***MyLC-2v*** | GCCAAGAAGCGGATAGAAGG | CTGTGGTTCAGGGCTCAGTC |
| ***β-MyHC*** | GCCAACACCAACCTGTCCAAGTTC | TGCAAAGGCTCCAGGTCTGAGGGC |
| ***Bmp2*** | ACACAGGGACACACCAACCAT | TGTGACCAGCTGTGTTCATCTTG |
| ***Bmp4*** | CTCCCAAGAATCATGGACTG | AAAGCAGAGCTCTCACTGGT |
| ***Fgf8*** | CAGCTCTACAGCCGCACCAGC | TGCTCTTGGCAATTAGCTTCC |
| ***Grem1*** | GCAACAGCCGCACTATCA | CCAAGTCGATGGATATGC |
| ***Wnt1*** | AAATCGCCCAACTTCTGCA | AATACCCAAAGAGGTCACAGC |
| ***Wnt3a*** | CTCCTCTCGGATACCTCTTAGTG | ATCCCTCTGCACAGGAGCGT |
| ***Wnt5a*** | CCCAGTCCGGACTACTGTGT | TTTGACATAGCAGCACCAGTG |
| ***Wnt7a*** | GACAAATACAACGAGGCCGT | GGCTGTCTTATTGCAGGCTC |
| ***Wnt11*** | GCCATGAAGGCCTGCCGTAG | GATGGTGTGACTGATGGTGG |
| ***SM-MyHC*** | GGATGCCACCACAGCCAAGTA | TGGTGTGGGTCCCTTCAGAGA |
| ***BrachyuryT*** | AAGGAACCACCGGTCATC | GTGTGCGTCAGTGGTGTGTAATG |
| ***Tbx6*** | ACCCGACCGTGTCTACATTCA | TGGTCTCAGGAAATCGGAAGG |
| ***Gapdh*** | TTCAACGGCACAGTCAAGG | CATGGACTGTGGTCATGAG |

**b. Primers for quantitative r**eal-time RT-PCR

| **Gene product** | **Sense** | **Anti-sense** |
| --- | --- | --- |
| ***Bmp2*** | ACACAGGGACACACCAACCAT | TGTGACCAGCTGTGTTCATCTTG |
| ***Bmp4*** | GACTTCGAGGCGACACTTCTA | GCCGGTAAAGATCCCTCATGTAA |
| ***Wnt3a*** | AATTTGGAGGAATGGTCTCTCGG | CAGCAGGTCTTCACTTCACAG |
| ***Gapdh*** | TGCGACTTCAACAGCAACTC | CTTGCTCAGTGTCCTTGCTG |
